# Association between comorbidities and differences in treatment decisions and outcomes in patients with colon or rectal cancer: a systematic review

**DOI:** 10.1136/bmjopen-2024-096492

**Published:** 2026-05-05

**Authors:** Abigail K Lloyd, Antonieta Medina-Lara, Stephen Birch, Katharine A Wallis, Anne Spencer

**Affiliations:** 1 Medical School, University of Exeter, Exeter, UK; 2 Faculty of Business, Economics & Law, The University of Queensland, Brisbane, Queensland, Australia; 3 General Practice Clinical Unit, Medical School, The University of Queensland, Brisbane, Queensland, Australia

**Keywords:** ONCOLOGY, Quality of Life, Health Care Costs

## Abstract

**Abstract:**

**Objectives:**

To systematically evaluate associations between comorbidities and differences in treatment decisions, outcomes, health-related quality of life (HRQoL), healthcare resource utilisation and costs, in patients with colon or rectal cancer.

**Design:**

Systematic review.

**Data sources:**

PubMed (Medline) and Embase databases were searched for studies published from January 2000 until January 2024.

**Eligibility criteria for selecting studies:**

We included articles that compared the presence and absence of comorbidities, evaluated multiple comorbid conditions or used the Charlson Comorbidity Index, or variations such as the Charlson-Deyo Index. Primary and secondary outcome measures included cancer treatments, outcomes (including complications from treatments, survival and mortality rates), HRQoL, healthcare resource use and costs.

**Data extraction and synthesis:**

Two independent reviewers used standardised methods to search, screen and code included studies. Risk of bias was assessed using the Joanna Briggs Institute checklists to ensure the quality of data. Findings were summarised narratively.

**Results:**

After duplicates were removed, 15 394 hits were screened and 31 studies were selected for inclusion in this systematic review. Comorbidities were associated with a lower likelihood of receiving treatment and lower survival rates and HRQoL, alongside a higher likelihood of complications following treatment, higher mortality rates and higher healthcare resource use. There were very limited studies that reported on HRQoL and resource use, and none reporting data directly relating to the impact of comorbidities on costs. These results were consistent across North America, Europe, Australia and New Zealand.

**Conclusions:**

For patients with colon and rectal cancer, comorbidities are associated with a lower likelihood of receiving treatments and poorer health outcomes. With global populations ageing, there is likely to be an increase in patients with colon and rectal cancer with comorbidities. Therefore, further research is necessary, especially to inform decisions regarding patient management and treatment, and to understand the implications on healthcare resource allocation, costs and HRQoL.

**STRENGTHS AND LIMITATIONS OF THIS STUDY**

- The study systematically searched and reviewed studies covering all aspects of the colon and rectal cancer treatment pathway and included a broad range of outcomes (eg, survival and mortality rates, health-related quality of life, resource use and costs).
- The review included the most common comorbidity measures, including presence and absence of disease.
- The critical appraisal tool applied in the review was not very sensitive and may have had limitations in assessing the quality of included studies.

## Introduction

 Colon and rectal cancer are, respectively, the fourth and seventh most prevalent cancers worldwide.^1^ In 2020, an estimated 1.15 million new cases of colon cancer and 700 000 new cases of rectal cancer were diagnosed, accounting for 6% and 3.8% of all new cancer cases, respectively,^2^ associated with approximately 575 000 and 340 000 deaths in 2020. The greatest incidence and mortality rates occur in countries with high incomes and medium or high human development index^3^; and recent trends show an increase in incidence rates is evident in North America, Europe, Australia and New Zealand, due to an increase in risk factors such as obesity and smoking.^4^

As populations in these high-income countries are ageing,^5 6^ and as both cancer and comorbidity diagnoses also increase with age,^7^ more patients are expected to be diagnosed with cancer and one or more other chronic conditions.^8^ These are referred to as comorbidities, defined as ‘any distinct additional clinical entity that has existed or that may occur during the clinical course of a patient who has the index disease under study’.^9^ Patients with cancer and comorbidities can be more challenging to manage as there are differences in time-to-diagnosis, treatment decisions, outcomes, healthcare resource utilisation and health-related quality of life (HRQoL). For instance, a review reported how comorbidities can either delay cancer diagnosis (through the masking of symptoms) or expedite cancer diagnosis (through increased presentation).^10^

In colon and rectal cancer research, previous systematic reviews on comorbidities generally focus on a singular cancer intervention, for example, adjuvant chemotherapy in patients with stage III colon cancer,^11^ or on one comorbidity, for example, diabetes.^12^ This systematic review addresses the current literature gap by evaluating associations between a wider set of comorbidity measures and differences in treatment decisions and outcomes in patients with colon or rectal cancer in high-income countries. Specifically, we assess similarities and differences in treatment decisions, outcomes (including complications, survival and mortality rates), HRQoL, healthcare resource utilisation and costs, associated with comorbidities, and whether these hold between different countries.

## Methods

### Study design

The study search strategy was designed to identify English language studies that evaluated the associations between comorbidities and differences in treatment decisions and outcomes in patients with colon or rectal cancer in individuals aged 18 years or over at the time of diagnosis, in high-income countries. Further details are available on PROSPERO (CRD42021227087).

### Search strategy

The search strategy was designed by an information specialist and included text and index terms for two databases, PubMed and Embase. The search strategy is included in the online supplemental materials (see pages 6–8). PubMed (Medline) and Embase databases were searched for studies published since January 2000 until January 2024 and duplicate studies were removed.

### Inclusion/exclusion criteria

The review included the most common comorbidity measures used from the most simple measures, such as types of comorbid conditions (CT), the number of comorbidities (N), and the presence of comorbidities (P) to weighted indices such as the Charlson Comorbidity Index (CCI),^13^ or variations or modified versions, including the Charlson-Deyo Index (CD).^14^ These weighted indices assign each patient a score based on the number and severity of comorbidities, so a patient with a higher score has a greater comorbid burden. The types of treatment included (surgery, chemotherapy, radiotherapy and combinations thereof) were defined by the WHO’s guidelines^15^ for early and late stage cancers.^16^ Studies were excluded if they did not include one of the included comorbidity measures or did not compare populations with vs without comorbidities or did not report the specified outcomes or were not available to the authors. Studies were also excluded if they were reporting findings from countries that were not high income, restricted the population group (eg, veterans) or did not cover relevant age groups (eg, focused on children), did not report the cancer sites or report the outcomes separately for colon and rectal. Finally, case reports, editorials, reviews and/or abstract-only publications were excluded.

### Screening process

In accordance with Preferred Reporting Items for Systematic Reviews and Meta-Analyses methodology,^17^ two investigators independently screened the studies. Abstracts were checked against the inclusion/exclusion criteria. The full text of the resulting studies was then checked against the inclusion and exclusion criteria before a final decision was made to retain or exclude them in the review.

### Data extraction

Data on study characteristics (author, year, title, journal, study design and country) participant characteristics (population, cancer site and stage, comorbidity measures and interventions) and methods used to control for cancer stage were extracted using a bespoke data extraction form. The data on treatment decisions that were extracted covered type, delays, dosage, receipt of treatment, type of resource use, admissions data, length of stay and costs. Outcome data were also extracted and included adverse events from treatments (e.g. complications), survival and mortality rates, and HRQoL. To ensure the reliability of the data extraction process, independent authors extracted data from a random sample.

### Data synthesis

A narrative synthesis provides a summary of the study characteristics, participant characteristics, comorbidity measure and methods used to control for cancer stage. The associations between comorbidities and differences in treatment decisions and outcomes for patients with colon and rectal cancer are synthesised into the following categories: (1) treatment decisions, (2) complications, (3) survival, (4) mortality and (5) HRQoL, healthcare resource use and costs. The synthesis is further divided into geographical regions (North America, Europe and Oceania) based on a study’s country of origin. Finally, this review includes a critical appraisal of the included studies using the corresponding Joanna Briggs Institute checklists to ensure the quality of data.^18^

### Patient and public involvement

Patients and/or the public were not involved in the design, or conduct, or reporting, or dissemination plans of this research.

## Results

### Included studies

**Figure 1:**
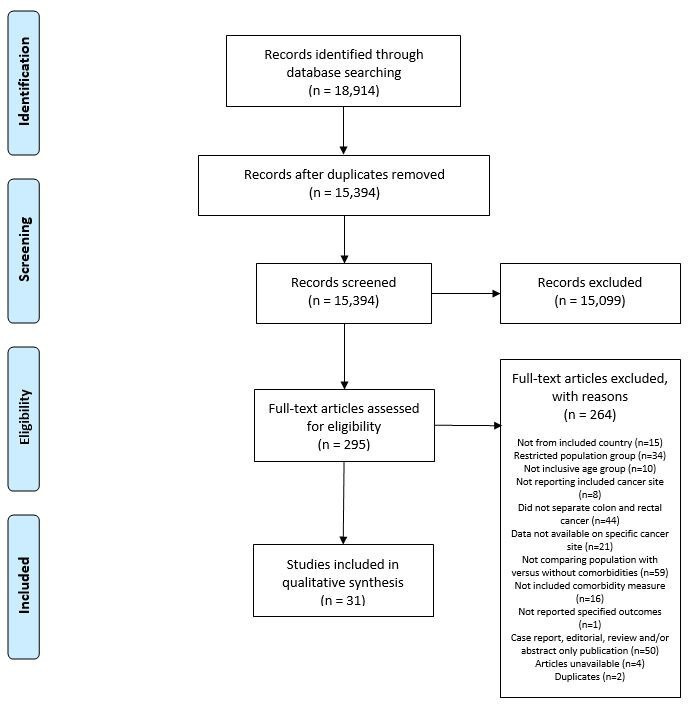
PRISMA flow diagram. The PRISMA process for screening studies with the relevant reasons for articles that were excluded and the final number of included studies. PRISMA, Preferred Reporting Items for Systematic Reviews and Meta-Analyses.

Electronic databases identified 18 914 studies and after duplicates were removed 15 394 abstracts were checked independently by two authors (AKL and AM-L) against the inclusion/exclusion. In total, 295 studies met the initial criteria and full studies were checked, with the final decision regarding eligibility being made by consensus. In this review, 31 studies were selected for inclusion (figure 1).

### Critical appraisal

The included studies were found to have a good adherence to the quality appraisal criteria; one cross-sectional study (1/1) and 18 cohort studies (18/30) fully met the quality criteria (see online supplemental material for summary checklist tables). All studies successfully measured both interventions and outcomes in clear, valid and reliable ways using appropriate statistical analysis. Most cohort studies clearly identified confounding variables (29/30), such as age, ethnicity, cancer stage and the route to diagnosis and strategies to deal with them (28/30). 18 cohort studies recruited similar groups from the same population, whereas 11 failed to make a clear distinction, and one utilised different population groups.

### Descriptive characteristics

Tables1  2 summarise the study and participant characteristics, as well as comorbidity measures and methods used to control for cancer stage, for colon and rectal cancer. The majority of the 22 studies that reported on colon cancer were from North America (USA n=10 and Canada n=3) followed by Europe (Netherlands n=5, Denmark n=2 and Finland n=1) and Oceania (New Zealand n=1).^19^,^40^ While the 16 studies that reported on rectal cancer were from North America (USA n=9) followed by Europe (Netherlands n=3, Denmark n=2, Finland n=1 and France=1, with none from Oceania.^2627 35^,^39 41^ Colon cancer studies had a sample size ranging from 348 to 360 846, compared with the rectal cancer studies that ranged from 143 to 55 181 patients. Most of the patients were male in colon cancer (range: 45.4%–53%), and rectal cancer studies (range: 56%–64.9%). The majority of studies used a weighted comorbidity index such as the CCI, CD or mCCI (20/22 colon and 16/16 rectal studies). Over 80% (19/22 colon and 13/16 rectal) of the studies clearly controlled for cancer stage. Further descriptive characteristic details are provided in the online supplemental materials.

**Table 1:** Summary characteristics of the colon cancer studies included in this systematic review

Author and date	Country	Study design	Treatments included	Sample size	Sex (% male)	Age*	Comorbidity measure†	Cancer stage
Reif de Paula 2023^19^	USA	Retrospective cohort study	Chemotherapy	11 847	45.96%	Median 63/77	CD	IV
Abdel-Rahman 2021^29^	Canada	Retrospective population-based study	Chemotherapy	2257	53	Mean 62/65	CCI	I–III
Clouth *et al* (2021)^32^	Netherlands	Population-based cohort study	Not reported	1489	52	Mean 70.5	N	I–III
Kellokumpu *et al* (2021)^39^	Finland	Retrospective population-based study	Surgical and chemotherapy and radiotherapy	968	48.7	Mean 70.7	ACCI	I–IV
Simon *et al* (2021)^20^	USA	Retrospective cohort study	Chemotherapy	5684	49.6	Median 70	CD	I–IV
Mukkamalla *et al* (2020)^21^	USA	Retrospective population-based cohort study	Chemotherapy	42 971	Not reported	18–64 to 75+	CD	II
Wegner *et al* (2020)^22^	USA	Retrospective population-based cohort study	Radiotherapy	23 325	51	Median 62	CD	IV
Xu *et al* (2019)^23^	USA	Retrospective cohort study	Surgical and chemotherapy	31 310	50.9	Median 62/63/73/74	mCCI	IV
Flemming *et al* (2017)^30^	Canada	Retrospective population-based cohort study	Surgical	4326	52	Median 71	CCI	I–IV
van den Broek *et al* (2017)^33^	Netherlands	Observational study	Chemotherapy	348	48.6	Median 73	CCI and N	III
Becerra *et al* (2016)^24^	USA	Retrospective multicentre cohort study	Surgical	360 846	47.5	Mean 69.4	mCCI	I–IV
Chandhoke *et al* (2016)^31^	Canada	Retrospective population-based cohort study	Chemotherapy	5289	51.4	Median 71	mCCI	II–III
Hsieh *et al* (2016)^25^	USA	Retrospective population-based cohort study	Chemotherapy	3180	50.8	<50 to 80+	CCI and P	III
Cakir *et al* (2015)^34^	Netherlands	Retrospective cohort study	Surgical	564	51	Mean 70	CT	Not reported
van Eeghen *et al* (2015)^35^	Netherlands	Cross-sectional single centre study	Not reported	184	56.5	<50 to 70+	CT	Not reported
Yeo *et al* (2015)^26^	USA	Retrospective population-based cohort study	Surgical	392	52.6	Median 71.6	CCI	I–IV
Ostenfeld *et al* (2013)^37^	Denmark	Retrospective population-based cohort study	Not reported	2493	49	Median 62.6	CCI	I–IV
Gooiker *et al* (2012)^36^	Netherlands	Retrospective cohort study	Not reported	5777	48	Median 72	CCI	Not reported
Bilimoria *et al* (2011)^27^	USA	Observational multicentre cohort study	Surgical	1407	47.8	<65 to 75+	CCI	I–III
Hines *et al* (2009)^28^	USA	Retrospective cohort study	Not reported	100 693	48.3	Median 73	CCI	I–III
Iversen *et al* (2009)^38^	Denmark	Population-based cohort study	Surgical	496	45.4	Median 66.9	CCI	I–IV
Sarfati *et al* (2009)^40^	New Zealand	Retrospective cohort study	Not reported	7970	47	Median 71/75/75	CCI	I–IV

* Age values are given as mean or median for the study, or for each subgroup (eg, subgroup 1/subgroup 2).

† The comorbidity measure used are CCI, ACCI, mCCI, CD, types of CT, N and P.

ACCI, age-adjusted CCI; CCI, Charlson Comorbidity Index; CD, Charlson-Deyo Index; CT, comorbid conditions; mCCI, modified version of CCI; N, number of comorbidities; P, presence of comorbidities.

**Table 2:** Summary characteristics of the rectal cancer studies included in this systematic review

Author and date	Country	Study design	Treatments included	Sample size	Sex (% male)	Age*	Comorbidity measure†	Cancer stage
Emile *et al* (2023)^41^	USA	Retrospective case–control cohort study	Surgical	55 181	60.9	Mean 61.2	CCI	I–III
Freund *et al* (2023)^42^	USA	Retrospective case–control cohort study	Surgical	9078	57.3	Mean 65/70	CCI	I
Kellokumpu *et al* (2021)^39^	Finland	Retrospective population-based cohort study	Surgical, chemotherapy and radiotherapy	511	64.8	Mean 67.9	ACCI	I–IV
Concors *et al* (2019)^43^	USA	Retrospective national cohort study	Surgical	8976	64.9	<50 to 70+	CD	IV
Wegner *et al* (2019)^45^	USA	Retrospective review	Chemoradiotherapy	7131	64	<65 to 65+	CD	I–III
El Amrani *et al* (2018)^49^	France	Prospective nationwide study	Surgical outcomes	45 569	61.5	<50 to 80+	CCI	I–IV
Wegner *et al* (2018)^44^	USA	Retrospective review	Radiotherapy	21 490	63	<60 to 60+	CD	II–III
Shahab *et al* (2017)^46^	USA	Retrospective population-based cohort study	Chemoradiotherapy	2891	60	Median 60.2	CD	II–III
Xu *et al* (2017)^47^	USA	Retrospective review	Chemotherapy	14 742	62.4	Median 56/60	CD	II-III
van Eeghen *et al* (2015)^35^	Netherlands	Cross-sectional single centre study	Not reported	143	58.7	Median 68	CCI	I–IV
Yeo *et al* (2015)^26^	USA	Retrospective population-based cohort study	Surgical	1539	56	Median 55.9	CCI	I–IV
Ostenfeld *et al* (2013)^37^	Denmark	Retrospective population-based cohort study	Not reported	2964	56	Median 69	CCI	Not reported
Gooiker *et al* (2012)^36^	Netherlands	Retrospective cohort study	Surgical outcomes	724	56.1	<65 to 75+	CCI	I–III
Bilimoria *et al* (2011)^27^	USA	Observational multicentre cohort study	Surgical	24 285	60.2	Median 66	CCI	I–III
Gort *et al* (2010)^48^	Netherlands	Retrospective population-based cohort study	Surgical	819	61.8	Median 68.4	mCCI	I–III
Iversen *et al* (2009)^38^	Denmark	Population-based cohort study	Surgical	5220	56	Median 68/74/75	CCI	I–IV

* Age values are given as mean or median for the study, or for each subgroup (eg, subgroup 1/subgroup 2).

† The comorbidity measure used are CCI, ACCI, mCCI, CD, types of CT, N and P.

ACCI, age-adjusted CCI; CCI, Charlson Comorbidity Index; CD, Charlson-Deyo Index; CT, comorbid conditions; mCCI, modified version of CCI; N, number of comorbidities.

### Outcome results

**Figure 2:**
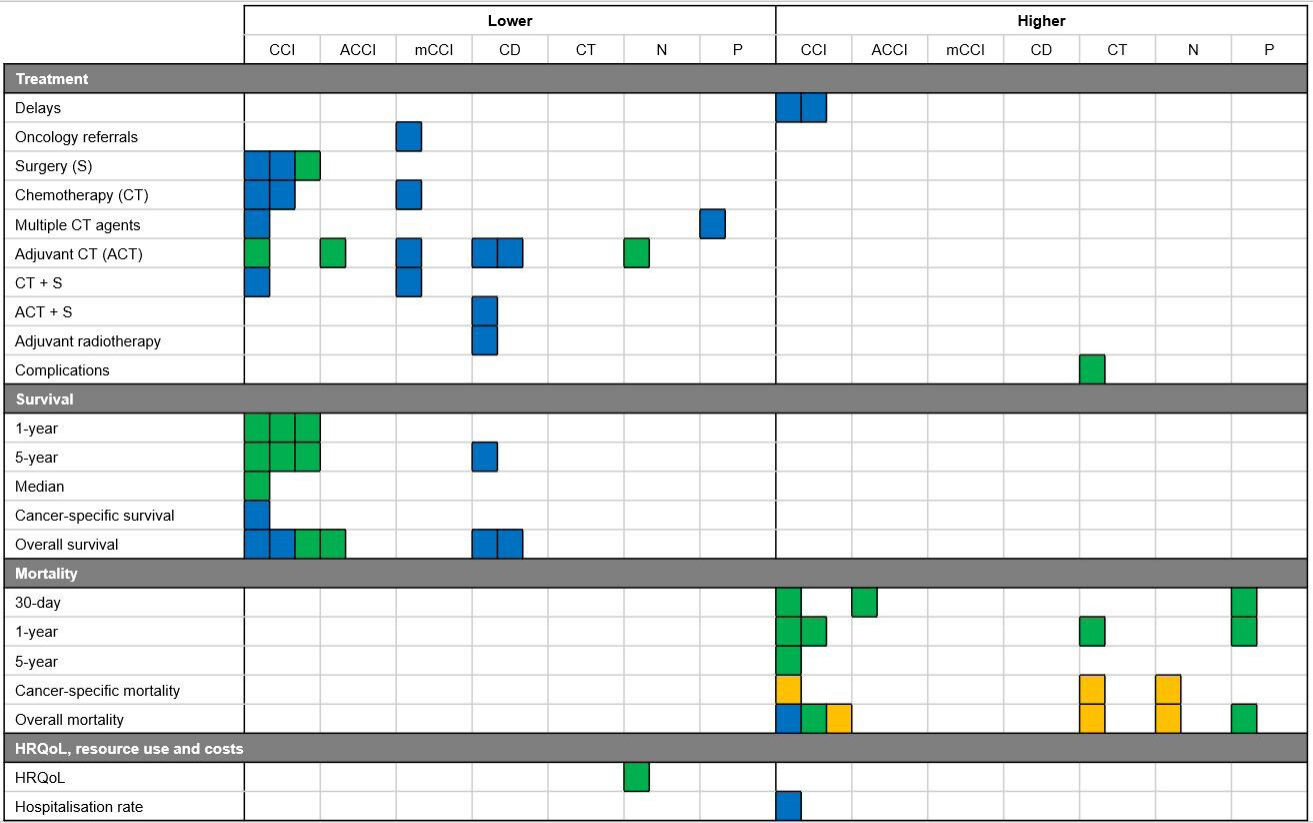
Summary of all of the studies’ results on the associations between comorbidities and treatment decisions and outcomes for patients with colon cancer. Each coloured square represents a study reporting that a comorbidity was associated with either an increased or decreased likelihood of the corresponding outcome. Note that a single study may report more than one outcome measure. The blue squares represent North American studies, green for European and orange for studies from Oceania. The results are divided into sections based on the comorbidity measure used: Charlson Comorbidity Index (CCI), age-adjusted CCI (ACCI), modified version of CCI (mCCI), Charlson-Deyo Index (CD), types of comorbid conditions (CT), the number of comorbidities (**N**) and the presence of comorbidities (**P**). HRQoL, health-related quality of life.

Results are considered statistically significant when studies reported results with p<0.05. The results are divided into five sections: treatment decisions, complications, survival, mortality and HRQoL, resource use and costs. Figures2  3 summarise comorbidities associated with either a higher or lower likelihood of each outcome, for colon and rectal cancer, respectively.

**Figure 3:**
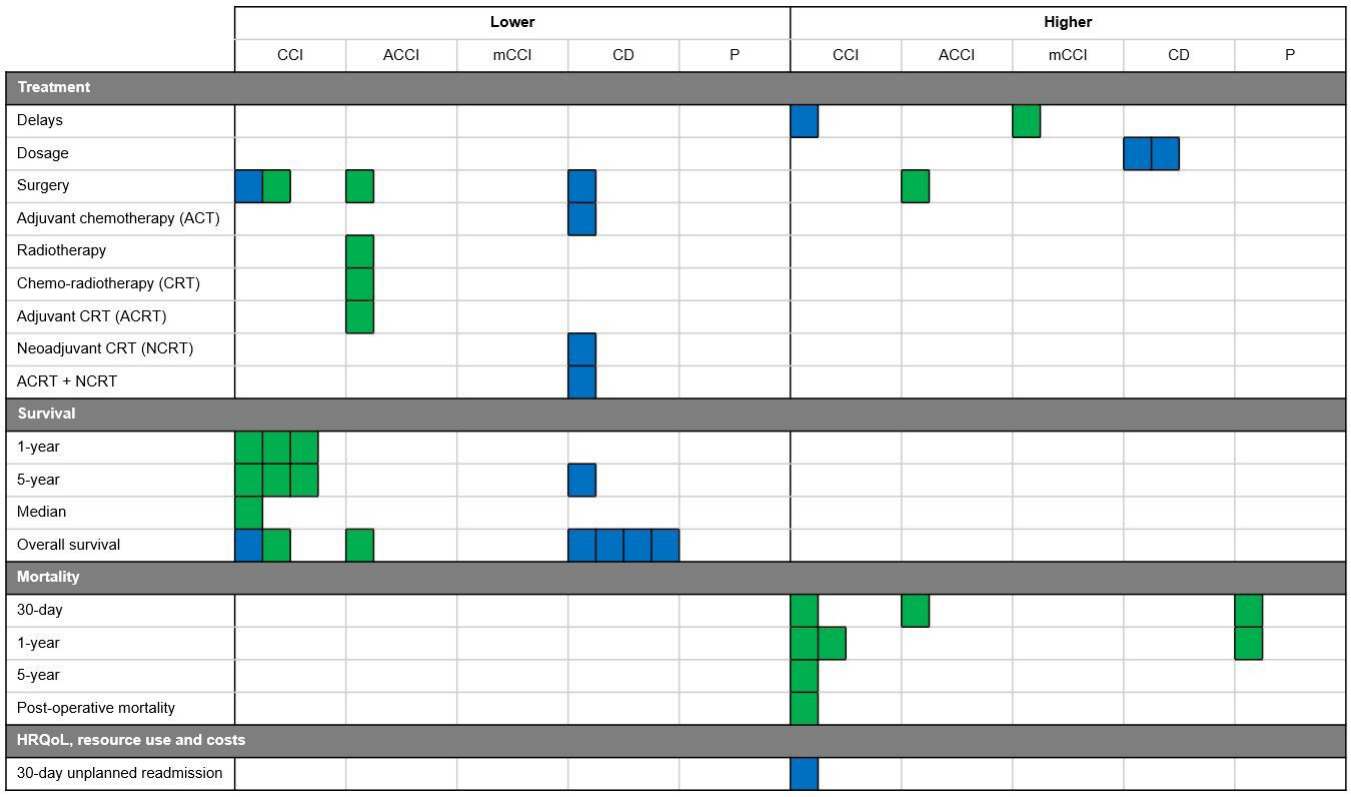
Summary of all of the studies’ results on the associations between comorbidities and treatment decisions and outcomes for patients with rectal cancer. Each coloured square represents a study reporting that a comorbidity was associated with either an increased or decreased likelihood of the corresponding outcome. Note that a single study may report more than one outcome measure. The blue squares represent North American studies and green for European. The results are divided into sections based on the comorbidity measure used: Charlson Comorbidity Index (CCI), age-adjusted CCI (ACCI), modified version of CCI (mCCI), Charlson-Deyo Index (CD) and the presence of comorbidities (**P**). HRQoL, health-related quality of life.

### Treatment decisions

#### North America

In colon cancer, 11 of 13 studies explored treatment decisions, from types of treatment and delays in treatment to treatment complications (as shown in figure 2) and reported differences in treatment decisions associated with comorbidities.^19^,^2730 31^ In the studies reporting significant differences in treatment delays, higher CCI scores were statistically significantly associated with delays in the time taken to start treatment of over 30 days,^27^ 42 days^30^ and 90 days.^30^ Higher CCI scores were associated with statistically significant lower proportions of patients receiving minimally invasive surgery (MIS),^26^ chemotherapy (CT) and lower use of multiple CT agents.^25^ Higher modified-CCI scores were statistically significantly associated with higher odds of receiving suboptimal, compared with optimal, lymphadenectomy,^24^ and lower Adjuvant CT (ACT),^31^ oncology referrals,^31^ ACT following referral^31^ and postoperative CT for patients with scores of 2 or more.^23^ Patients with higher CCI scores were less likely to receive surgery, CT and surgery in conjunction with CT.^23^ While patients with higher CD scores were statistically significantly less likely to receive ACT,^19 21^ ACT following surgery^20^ and adjuvant radiotherapy.^22^ Finally, the absence of comorbidities was associated with statistically significant higher use of multiple chemotherapy agents during therapy.^25^

In rectal cancer studies, 7 of 9 studies explored treatment decisions and reported differences in treatment decisions associated with comorbidities.^2627 43^,^47^ In studies reporting significant differences, one study reported higher CCI scores were associated with delays in the time-to-treatment onset of over 30 days, which was significant for patients with scores greater than 2.^27^ Higher CCI scores were linked to lower rates of MIS,^26^ while CD scores were associated with statistically significant lower rates of combined proctectomy and hepatectomy surgeries,^43^ ACT,^47^ neoadjuvant chemoradiotherapy,^46^ and a combination of adjuvant and neoadjuvant chemoradiotherapy.^46^ Additionally, two studies reported that for patients with CD scores of two or more, there were statistically significant differences in the administration of radiotherapy dosages.^44 45^ Patients with higher CD scores were more likely to receive dose-escalated therapy (defined as any dose >54)^45^ and intensity-modulated radiotherapy,^44^ compared with conventional dosage treatments.

#### Europe

Three of the eight colon cancer studies explored treatment decisions and reported differences in treatment decisions associated with comorbidities.^33 38 39^ In the studies reporting significant differences, higher CCI scores were linked with higher proportions of patients receiving no surgery, a lower resection rate, yet no difference in local procedure rates.^38^ Higher CCI scores,^33^ age-adjusted CCI (ACCI) scores^39^ and the number of comorbidities^33^ were statistically significantly associated with lower administration of ACT; but higher ACCI scores were associated with no significant differences in the rates of surgical resection, surgery type or emergency surgery.^39^

For rectal cancer studies, three of seven studies explored treatment decisions and reported differences in treatment decisions associated with comorbidities.^38 39 48^ Higher CCI scores were associated with lower proportions of patients receiving surgery and lower resection rates, but a slightly higher local procedure rate.^38^ The study investigating the time from diagnosis to undergoing surgical procedures found that the time-to-treatment onset was statistically significantly related to comorbidities: the likelihood of early treatment was almost two times lower for patients with a mCCI of two or more.^48^ Higher ACCI scores were also statistically significantly associated with higher rates of local surgical procedures, but lower rates of major elective surgery, emergency surgery, radiotherapy and chemoradiotherapy.^39^ When grouped by cancer stage, patients with higher ACCI scores were less likely to receive ACT, but this was only statistically significant in patients with stage II cancer.

### Treatment complications

#### Europe

In colon cancer study, one of the eight studies explored treatment complications and reported that patients with pulmonary comorbidities were statistically significantly more likely to suffer from pneumonia and undergo reoperation following their initial treatment.^34^

### Survival

#### North America

For colon cancer studies, 5 in 13 studies explored survival and found associations between comorbidities and lower survival rates.^19 21 22 29 30^ For studies reporting differences, higher CCI scores were linked with statistically significantly lower overall survival and cancer-specific survival in one study^30^ and non-significant lower overall survival in another study.^29^ Additionally, higher CD scores were associated with statistically significantly lower 5-year survival^21^ and overall survival.^19 22^

In rectal cancer studies, six in nine studies explored survival and found associations between comorbidities and lower survival rates.^42^,^47^ For studies reporting significant differences, higher CD scores were statistically significantly associated with lower overall survival^43^,^46^ and 5-year survival.^47^ Higher CCI scores were statistically significantly linked to lower overall survival.^42^

#### Europe

In colon cancer studies, four in eight studies explored survival and reported associations between comorbidities and lower survival rates.^3537^,^39^ For studies reporting significant differences, higher CCI scores were associated with lower median,^35^ 1-year^35 37 38^ and 5-year survival.^35 37 38^ Overall survival was statistically significantly lower in association with higher CCI^35^ and ACCI scores.^39^

In rectal cancer studies, four in seven studies explored survival and reported associations between comorbidities and lower survival rates.^3537^,^39^ For studies reporting significant differences, higher CCI scores were associated with lower median,^35^ 1-year,^35 37 38^ 5-year^35 37 38^ and overall survival.^35^ ACCI scores were associated with statistically significant lower overall survival.^39^ Additionally, lower disease-free survival was statistically significantly linked to the higher time taken before treatment, which in turn was statistically significantly associated with higher comorbidity scores; however, data on survival rates were not specifically linked to comorbidities.^48^

### Mortality

#### North America

For colon cancer study, only 1 in 13 studies explored mortality and reported increasing CCI scores were associated with statistically significantly higher overall mortality rates.^28^

#### Europe

For colon cancer studies, four in eight studies explored mortality and reported associations between comorbidities and higher mortality rates.^35 36 38 39^ For studies reporting significant differences, higher CCI scores were associated with statistically significant higher 30-day,^36^ 1-year overall^36^ and 1-year excess mortality^36^ and non-statistically significant higher 1-year relative mortality,^38^ 5-year relative mortality^38^ and overall mortality.^35^ Higher ACCI scores were statistically significantly associated with higher 30-day mortality,^39^ while the presence of hypertension, vascular disease, kidney disease and neurological disease was associated with statistically significant higher 1-year excess mortality.^36^ Finally, the presence of comorbidities was statistically significantly associated with higher 30-day, 1-year excess and 1-year overall mortality rates.^36^

In rectal cancer studies, four in seven studies explored mortality and reported associations between comorbidities and higher mortality rates.^36 38 39 49^ For studies reporting significant differences, higher CCI scores were associated with statistically significant higher postoperative,^49^ 30-day,^36^ 1-year overall^36^ and 1-year excess mortality^36^ and non-significantly higher 1-year relative mortality^38^ and 5-year relative mortality.^38^ Only ACCI scores of four or greater were associated with higher 30-day mortality, but this was not statistically significant.^39^ Finally, the presence of comorbidities was statistically significantly associated with higher 30-day, 1-year excess and 1-year overall mortality.^36^

#### Oceania

In the one colon cancer study included in our review in Oceania, higher CCI scores, the number of comorbidities and comorbid condition types were all associated with statistically significant higher all-cause mortality and non-significantly higher cancer-specific mortality.^40^

### HRQoL, resource use and costs

#### North America

In the colon cancer study, only 1 in 13 studies explored resource use and found that higher CCI scores were statistically significantly associated with a higher number of hospitalisation days during adjuvant chemoradiotherapy and higher hospitalisation risk.^29^ For the rectal cancer study, only one in nine studies explored resource use and found that higher CCI scores were statistically significantly associated with higher likelihood of 30-day unplanned hospital re-admission after surgery.^41^

#### Europe

In colon cancer, only one in eight studies explored patients’ HRQoL. This study used the European Organisation for Research and Treatment of Cancer Quality of Life Questionnaire,^50^ a disease-specific cancer questionnaire, and found that higher number of comorbidities was statistically significantly associated with lower HRQoL.^32^

## Discussion

### Principal findings

This section gives an overview of the principal findings of the review and reports in parentheses the number of studies reporting significant differences out of the total number of studies assessing each outcome, that is (‘total significant results/total studies measuring each outcome’).

Colon cancer studies reported comorbidities were associated with significantly higher likelihood of delays between diagnosis and starting treatment (2/2), but a lower likelihood of receiving treatment (12/12) and higher likelihood of complications following treatment (1/1). Survival rates (8/9) and HRQoL (1/1) were lower (although evidence for HRQoL is limited), while mortality rates (4/6) and hospitalisation risks (1/1) were higher. There were no studies that reported on resource use or costs.

Fewer studies reported on rectal cancer, but also found that comorbidities were associated with significant delays in receiving treatment (2/2) and a lower likelihood of receiving treatment (6/8). Interestingly, even among studies that reported lower overall receipt of treatment, higher rates of local surgical procedures were sometimes reported (2/8). Higher doses of radiotherapy treatments were also reported (2/2). Survival rates were lower (10/10), while mortality rates (2/4) and likelihood of unplanned re-admissions (1/1) were higher. There were no studies that reported on HRQoL, costs or resource use.

There was only one study from Oceania, so it is hard to draw any comparisons except those between North America and Europe. Those results were consistent, with only two European studies on rectal cancer reporting outliers (eg, higher local surgical procedure rates). Most studies used weighted indices to measure comorbidities, with CCI being the most common across all geographic regions. Only European studies used the ACCI, while only studies carried out in the USA used the CD Index.

### Key results

#### Treatments

Studies consistently showed that patients with cancer and comorbidities were more likely to experience delays in treatment and were less likely to receive cancer treatment than patients without comorbidities. While waiting times for treatments have increased over recent decades,^27^ comorbidities were associated with a longer time between diagnosis and surgery for patients with both colon and rectal cancer.^27 30 48^ Such delays have been attributed to delayed diagnosis^10^ or differential patient management; for example, fewer referrals to oncologists were made in a vignette-based study when the patient had severe comorbidities.^51^ The delay could also be the result of slower decision making as comorbidities require addressing prior to treatment to minimise potential risks, which often involves a multidisciplinary approach.^30^ This is in line with one study in this review that showed patients with colon cancer and comorbidities were more likely to suffer from adverse effects postsurgery and increased risk of reoperation.^34^

Most studies, for both colon and rectal cancer, showed patients with comorbidities were less likely to receive curative cancer treatment. Two studies reported higher local surgical procedure rates for colon and rectal cancer, but this difference was small, especially in comparison to the large reductions in elective, emergency and overall surgery they also reported.^38 39^ Overall, the results showed comorbidities were associated with lower receipt of surgery, chemotherapy, radiotherapy and combinations thereof, which is consistent with current literature.^52^ The primary explanations for differential treatments are due to disparities in patient and clinician decision making. Patients may discontinue or refuse treatment due to increased adverse effects and reduced quality of life,^53^ while clinicians may be concerned that comorbidities will exacerbate treatment toxicity, reduce effectiveness or that the life expectancy of the patient is insufficient to justify treatment.^52 54^ Interactions between comorbidities and cancer are often overlooked in clinical guidelines which generally adopt a ‘single-disease’ approach.^55^ Comorbid patients, therefore, are less likely to receive treatments consistent with current guidelines, and as the guidelines were not developed for comorbid patients further study is needed to update them.

The cause and degree of the impact of comorbidities on treatment decisions and timings were not consistently recorded, making it challenging to draw further conclusions. For instance, it is unclear if patients with comorbidities were managed differently due to clinicians deeming comorbidities to be inhibitive, or because comorbidity treatment was prioritised. There is, overall, a lack of high-level evidence relating to the impact of comorbidities on cancer treatment due to the common exclusion of comorbid patients from randomised controlled trials.^52^

#### Survival

All studies from North America and Europe that reported survival outcomes showed that comorbidities were associated with lower survival rates for patients with colon and rectal cancer. The incidence of colon and rectal cancer is highest in Australia, New Zealand, Europe and North America.^4^ They show some variation in survival rates, but differences are most pronounced between colon and rectal cancer.^56^ One-year age-standardised survival for colon cancer ranges from 77.1% (UK) to 87.5% (Australia), whereas rectal cancer survival ranges from 84.8% (UK) to 90% (Australia). Across the geographic regions included in this review, 1-year survival was higher for rectal cancer than colon cancer in all countries with differences ranging from 2.5% (Australia) to 7.7% (UK). Survival rates were similar (<1% difference) between colon and rectal cancer in Australia, Canada and Ireland, but for all other countries in the included geographic regions, rectal cancer had higher 5-year net survival rates. Survival differences internationally have been explained due to differences in when countries implemented screening programmes, differential diagnostic guidelines, time-to-treatment and treatment adherence.^56^ Lower survival rates for colon cancer compared with rectal cancer are not yet fully understood. It could be associated with greater increases in incidence among younger age groups, but this increase is less pronounced among patients with colon cancer.^57^ Interestingly, a recent study has shown that the presence of comorbidities is significantly higher in patients with colon cancer (77.4%) compared with patients with rectal cancer (74.2%).^58^ These associations between comorbidities and cancer survival warrant further research, especially given the increasing incidence of comorbidities among patients with cancer.^59^ Nevertheless, current global trends show survival rates continue to increase over time.^60^

#### Mortality

All studies that reported data on mortality showed colon and rectal cancer mortality rates were higher for patients with comorbidities. This is important as comorbidities among patients with cancer are increasing, with continued increases of 0.54% predicted annually.^59^ The highest incidence of comorbidities in patients with cancer is in Europe and North America,^59^ which, along with Australia and New Zealand, also have the highest incidence of colon and rectal cancer.^4^ The higher rates in these geographic regions are likely a result of increased risk factors including obesity, smoking and alcohol consumption.^57^ The age-standardised incidence rates of both colon and rectal cancers are similar across geographic regions in this review.^3^ Colon cancer incidence has increased over time in Europe, but decreased in North America and Oceania. Rectal cancer incidence has decreased overall in the included regions. This has resulted in similar mortality rates across these countries, with an average annual percentage change of −1.18% to −2.45% in North America, +0.21% to −3.64% in Europe and −1.66% to −2.74% in Oceania. Interestingly, the mortality rates for colon and rectal cancer are predicted to diverge in the future.^57^ In the included regions, mortality rates for colon cancer are predicted to decrease while rectal cancer mortality rates are projected to increase. These differences could be due to more pronounced increases in rectal cancer incidence in younger age groups, but the underlying reasons are not fully understood.^3 57^ Even with colon cancer mortality rates predicted to decrease, due to population growth, the absolute number of deaths from both colon and rectal cancer will continue to increase.^57^ Our results are consistent with these trends, and with ageing populations and an increase in comorbidity prevalence,^59^ associations between comorbidities and cancer mortality should be explored in future research. For instance, while research has shown that patients with cancer with comorbidities have higher mortality rates, there remains insufficient evidence distinguishing between patients that died from cancer and those that died from their comorbidity.

#### HRQoL, resource use and costs

This review has highlighted insufficient levels of research in these areas. For colon cancer, there were only a few studies reporting HRQoL, costs or resource use in North America and Europe. However, these studies examined either resource use and costs or HRQoL, but did not assess them together. For rectal cancer, no study reported these measures. HRQoL research is necessary to improve our understanding of patients’ quality of life. How comorbidities influence this is important to inform clinical decisions and facilitate the tailoring of individual treatments to maximise overall health. Additionally, for economic evaluation and policy recommendations, it is important to measure HRQoL alongside resource use and costs to enable comparisons across different disease groups and treatments. The lack of data on HRQoL and healthcare resource utilisation restricts the understanding of the economic impact of colon and rectal cancer on patients, families and healthcare systems. This information is essential to inform decisions on the management of patients and allocation of healthcare resources, and without it, policy makers are unable to develop credible guidelines informed by cost-effectiveness analyses.

### Strengths and weaknesses

This systematic review searched for studies covering all aspects of the colon and rectal cancer pathway and included a range of outcomes. To enable this broad scope, restrictions were placed, and potentially relevant studies were excluded, based on two criteria: comorbidity measures and country of origin. First, in relation to comorbidity measures, we only included studies that utilised the most common measurements. This allowed for a large evidence base to be included while reducing heterogeneity. Second, due to substantial differences in healthcare resource availability globally, it was not feasible to include all countries. Various methodologies for classifying countries based on healthcare systems were considered, but results were inconsistent.^61 62^ Therefore, as a determining factor in variability between treatment plans is resource availability, to reduce the heterogeneity between studies, only high-income countries were included. Our results did not differ between different comorbidity measures or countries, so these limitations are unlikely to have altered the conclusions of this review. However, a weakness of our study is that the databases were searched from January 2020 to January 2024, and we did not have capacity to update the searches due to the time-limited availability of funding. Another weakness of the review is that the critical appraisal tool that we used was not very sensitive and all studies scored eight or more out of the 10-point criteria. The Joanna Briggs Institute is a well-respected critical appraisal tool, but in this application, determining whether the two groups were similar and recruited from the same population was often unclear and could not easily be assessed in these types of studies.

### Policy recommendations

This systematic review has highlighted that, in patients with colon and/or rectal cancer, a higher comorbidity score is significantly associated with patients being less likely to receive treatment and poorer outcomes including higher mortality and lower survival. Unfortunately, given the limited number of studies reporting data on HRQoL, healthcare resource utilisation and costs, the broader impact of comorbidities on healthcare systems remains unclear. Therefore, policy makers lack suitable evidence to inform decisions on optimising the health of comorbid patients with colon and rectal cancer.

### Research recommendations

Further research is needed to understand the impact of comorbidities on HRQoL to enable policy makers to estimate both survival and HRQoL changes. This is essential to provide a broader understanding of the impacts of treatments on patients. Information is also necessary on the reasons for treatment delays and disparities between patients due to comorbidities. Additionally, to better understand the differences in treatment decisions and patient management, research focused on differential healthcare resource utilisation and costs is required. Finally, in order to evaluate causation in such research, future studies need to control for unobserved confounding. In this review the majority of studies only control for observed confounding variables; thus, results are limited to assessing associations between comorbidities and cancer.

## Supplementary material

10.1136/bmjopen-2024-096492 online supplemental file 1

## Data Availability

No data are available.
