# Supplementary material for: Association between comorbidities and differences in treatment decisions and outcomes in patients with colon or rectal cancer: a systematic review
Source: BMJ Open. 2026 May 5;16(5):e096492. doi: 10.1136/bmjopen-2024-096492 (PMC13150882; doi:10.1136/bmjopen-2024-096492)
Supplement: online supplemental file 1 [file bmjopen-16-5-s001.docx]

**SUPPLEMENTARY MATERIALS**

**Critical appraisals**

Table S1. Responses to the critical appraisal JBI checklist questions of the cross-sectional study

| Author & date | Were the criteria for inclusion in the sample clearly defined? | Were the study subjects and the setting described in detail? | Was the exposure measured in a valid and reliable way? | Were objective, standard criteria used for measurement of the condition? | Were confounding factors identified? | Were strategies to deal with confounding factors stated? | Were the outcomes measured in a valid and reliable way? | Was appropriate statistical analysis used? | Overall appraisal |
| --- | --- | --- | --- | --- | --- | --- | --- | --- | --- |
| van Eeghen 2015  (1) | Yes | Yes | Yes | Yes | Yes | Yes | Yes | Yes | Include |

Table S2. Responses to the critical appraisal JBI checklist questions of the cohort studies

| Author & date | Were the two groups similar and recruited from the same population? | Were the exposures measured similarly to assign people to both exposed and unexposed groups? | Was the exposure measured in a valid and reliable way? | Were confounding factors identified? | Were strategies to deal with confounding factors stated? | Were the groups/participants free of the outcome at the start of the study (or at the moment of exposure)? | Were the outcomes measured in a valid and reliable way? | Was the follow up time reported and sufficient to be long enough for outcomes to occur? | Was follow up complete, and if not, were the reasons to loss to follow up described and explored? | Were strategies to address incomplete follow up utilised? | Was appropriate statistical analysis used? | Overall appraisal |
| --- | --- | --- | --- | --- | --- | --- | --- | --- | --- | --- | --- | --- |
| Emile 2023 (2) | Unclear | Yes | Yes | Yes | Yes | Yes | Yes | Yes | Yes | N/A | Yes | Include |
| Freund 2023 (3) | Yes | Yes | Yes | Yes | Yes | Yes | Yes | Yes | Yes | N/A | Yes | Include |
| Reif de Paula 2023 (4) | Yes | Yes | Yes | Yes | Yes | Yes | Yes | Yes | Yes | N/A | Yes | Include |
| Abdel-Rahman 2021(5) | Yes | Yes | Yes | Yes | Yes | Yes | Yes | Yes | Yes | N/A | Yes | Include |
| Clouth 2021(6) | Unclear | Yes | Yes | Yes | Yes | Yes | Yes | Yes | Yes | N/A | Yes | Include |
| Kellokumpu 2021(7) | Yes | Yes | Yes | Yes | Yes | Yes | Yes | Yes | Yes | N/A | Yes | Include |
| Simon 2021(8) | Yes | Yes | Yes | Yes | Yes | Yes | Yes | Yes | Yes | N/A | Yes | Include |
| Mukkamalla 2020(9) | Unclear | Yes | Yes | Yes | Yes | Yes | Yes | Yes | Yes | N/A | Yes | Include |
| Wegner 2020(10) | Unclear | Yes | Yes | Yes | Yes | Yes | Yes | Yes | Yes | N/A | Yes | Include |
| Concors 2019(11) | Unclear | Yes | Yes | Yes | Yes | Yes | Yes | Yes | Yes | N/A | Yes | Include |
| Wegner 2019(12) | Yes | Yes | Yes | Yes | Yes | Yes | Yes | Yes | Yes | N/A | Yes | Include |
| Xu 2019(13) | Yes | Yes | Yes | Yes | Yes | Yes | Yes | Yes | Yes | N/A | Yes | Include |
| El Amrani 2018(14) | Yes | Yes | Yes | Yes | Yes | Yes | Yes | Yes | Yes | N/A | Yes | Include |
| Wegner 2018(15) | Yes | Yes | Yes | Yes | Yes | Yes | Yes | Yes | Yes | N/A | Yes | Include |
| Flemming 2017(16) | Unclear | Yes | Yes | Yes | Yes | Yes | Yes | Yes | Yes | N/A | Yes | Include |
| Shahab 2017(17) | Yes | Yes | Yes | Yes | Yes | Yes | Yes | Yes | Yes | N/A | Yes | Include |
| van den Broek 2017(18) | Unclear | Yes | Yes | Yes | Unclear | Yes | Yes | Yes | Yes | N/A | Yes | Include |
| Xu 2017(19) | Unclear | Yes | Yes | Yes | Yes | Yes | Yes | Yes | Yes | N/A | Yes | Include |
| Becerra 2016(20) | Unclear | Yes | Yes | Yes | Yes | Yes | Yes | Yes | Yes | N/A | Yes | Include |
| Chandhoke 2016(21) | Yes | Yes | Yes | Yes | Yes | Yes | Yes | Yes | Yes | N/A | Yes | Include |
| Hsieh 2016 (22) | Yes | Yes | Yes | Yes | Yes | Yes | Yes | Yes | Yes | N/A | Yes | Include |
| Cakir 2015(23) | No | Yes | Yes | Unclear | Unclear | Yes | Yes | Yes | Yes | N/A | Yes | Include |
| Yeo 2015(24) | Yes | Yes | Yes | Yes | Yes | Yes | Yes | Yes | Yes | N/A | Yes | Include |
| Ostenfeld 2013(25) | Yes | Yes | Yes | Yes | Yes | Yes | Yes | Yes | Yes | N/A | Yes | Include |
| Gooiker 2012(26) | Yes | Yes | Yes | Yes | Yes | Yes | Yes | Yes | Yes | N/A | Yes | Include |
| Bilimoria 2011(27) | Yes | Yes | Yes | Yes | Yes | Yes | Yes | Yes | Yes | N/A | Yes | Include |
| Gort 2010(28) | Yes | Yes | Yes | Yes | Yes | Yes | Yes | Yes | Yes | N/A | Yes | Include |
| Hines 2009(29) | Unclear | Yes | Yes | Yes | Yes | Yes | Yes | Yes | Yes | N/A | Yes | Include |
| Iversen 2009(30) | Yes | Yes | Yes | Yes | Yes | Yes | Yes | Yes | Yes | N/A | Yes | Include |
| Sarfati 2009(31) | Unclear | Yes | Yes | Yes | Yes | Yes | Yes | Yes | Yes | N/A | Yes | Include |

**Study and Participant Characteristics**

Study characteristics

*North America*

There were 13 *colon* cancer studies from North America (USA (n=10) (4, 8-10, 13, 20, 22, 24, 27, 29) and Canada (n=3) (5, 16, 21)). Of these twelve studies were retrospective; six were retrospective population-based cohort studies (9, 10, 16, 21, 22, 24), four were retrospective cohort studies (4, 8, 13, 29), one retrospective population-based study (5), and one a retrospective multi-centre cohort study (20). The remaining study was an observational multi-centre cohort study (27). The interventions studied included chemotherapy (n=6) (4, 5, 8, 9, 21, 22), surgical interventions (n=4) (16, 20, 24, 27), surgery and chemotherapy (n=1) (13), radiotherapy (n=1) (10), and one study did not report data on intervention (29).

There were nine *rectal* cancer studies from the USA (2, 3, 11, 12, 15, 17, 19, 24, 27), all of which were retrospective studies, with two specifically reporting as population-based cohort studies (17, 24), two case-controlled cohort studies (2, 3), one as a national cohort study (11), and one multi-centre cohort study (27). The interventions studies included surgery (n=5) (2, 3, 11, 24, 27), chemo-radiotherapy (n=2) (12, 17), chemotherapy (n=1) (19), and radiotherapy (n=1) (15).

*Europe*

There were eight *colon* cancer studies from Europe (Netherlands (n=5) (1, 6, 18, 23, 26), Denmark (n=2) (25, 30), and Finland (n=1) (7)); of these five were retrospective studies, two were retrospective cohort studies (23, 26), and two were retrospective population-based cohort studies (7, 25). Two studies were population-based cohort studies (6, 30), one was a cross-sectional single-centre study (1), and one was an observational study (18). The interventions studies included surgery (n=2) (23, 30), chemotherapy (n=1) (18), and surgery, chemotherapy and radiotherapy (n=1) (7), whilst the remaining four studies did not report data on intervention (1, 6, 25, 26).

There were seven *rectal* cancer studies from Europe (Netherlands (n=3) (1, 26, 28), Denmark (n=2) (25, 30), Finland (n=1) (7), and France (n=1) (14)); of these three were retrospective population-based cohort studies (7, 25, 28), one was a retrospective cohort study (26), one population-based cohort study (30), one prospective nationwide study (14), and one cross-sectional single-centre study (1). The interventions studied included surgery (n=4) (14, 26, 28, 30), (with two specifically focused on outcomes after surgery (14, 26)), surgery, chemotherapy and radiotherapy (n=1) (7), whilst the remaining two studies did not report data on intervention (1, 25).

*Oceania*

Only one retrospective cohort *colon* cancer study, that did not report data on interventions, from New Zealand was included in this review (31). There were no studies on *rectal* cancer from Oceania included in this review.

Participant characteristics

*North America*

Five *colon* cancer studies included participants with cancer stages I-IV (8, 16, 20, 24, 29), whilst five studies only included participants with one cancer stage; stage II (n=1) (9), stage III (n=1) (22), or stage IV (n=3) (4, 10, 13). Two studies included participants with cancer stages I-III (5, 27), and one included stages II-III (21). These 13 studies had a sample size range of 496 to 360,846 with a mean of 45,747. The majority of participants were not male (45.8%; range 45.4% to 53%). Nine studies reported the median age of participants ranging from 62 to 77 years (4, 8, 10, 13, 16, 21, 24, 27, 29), and two studies reported the mean age ranging from 62 to 69 years (5, 20). The remaining two studies used age categories ranging from under 65 to over 75 (9), and under 50 to over 80 (22).

Three *rectal* cancer studies included participants with cancer stages II-III (15, 17, 19), three studies included stages I-III (2, 12, 27), and one included all cancer stages (I-IV) (24). Two studies only included participants with one cancer stage; stage I (n=1) (3), and stage IV (n=1) (11). These seven studies had a sample size range of 1,539 to 55,181 with a mean of 16,146. The majority of participants were male (60.9%; range 56% to 64.9%). Four studies reported the median age of participants ranging from 55.9 to 66 years (17, 19, 24, 27), two studies reported the mean age of participants ranging from 61.2 to 69.9 years (2, 3), two studies listed participants as either under of over aged 60 (15), and under of over 65 (12), and the remaining study reported age categories ranging from under 50 to over 70 (11).

*Europe*

Three *colon* cancer studies included participants with cancer stages I-IV (1, 7, 30), two studies included stages I-III (6, 26), one study restricted participants to stage III (18), and two studies did not report cancer stage (23, 25). These eight studies had a sample size range of 348 to 7,970 with a mean of 2364. The majority of participants were not male (49.5%; range 47% to 52.6%). Four studies reported the median age of participants ranging from 71 to 75 years (1, 18, 25, 30), and three studies reported the mean age ranging from 70 to 70.7 years (6, 7, 23). The remaining study used age categories ranging from under 65 to over 75 (26).

Four *rectal* cancer studies included participants with cancer stages I-IV (1, 7, 14, 30), two studies included stages I-III (26, 28), and the remaining study did not report cancer stage (25). These five studies had a sample size range of 143 to 45,569 with a mean of 7,993. The majority of participants were male (59.3%; range 56% to 64.8%). Four studies reported the median age of participants ranging from 68 to 75 years (1, 25, 28, 30), and one study reported the mean age of participants as 67.9 (7). Two studies used age categories ranging from under 50 to over 80 (14), and under 65 to over 75 (26).

*Oceania*

The *colon* cancer study included participants with cancer stages I-IV (31). It had a sample size of 776, with the majority of participants being male (51.8%). Age was reported used categories ranging from under 25 to over 75.

Comorbidity measures

*North America*

All 13 *colon* cancer studies utilised a weighted comorbidity index; six used the CCI (5, 16, 22, 24, 27, 29), four used the Charlson-Deyo Index (4, 8-10), and three used an unspecified modified CCI (13, 20, 21). One study also recorded the presence or absence of comorbidities (22).

All nine *rectal* cancer studies utilised a weighted comorbidity index, five used the Charlson-Deyo Index (11, 12, 15, 17, 19), and four used the CCI (2, 3, 24, 27).

*Europe*

Five *colon* cancer studies used the CCI (1, 18, 25, 26, 30), (with one also recording the number of comorbidities (18),) and one used the age-adjusted CCI (ACCI) (7). One study recorded the type of comorbid condition (23), and one reported the number of comorbidities (6).

All seven *rectal* cancer studies utilised a weighted comorbidity index; five studies used the CCI (1, 14, 25, 26, 30), one study used the ACCI (7), and one used an unspecified modified version (28).

*Oceania*

Comorbidities in the *colon* cancer study were recorded using the CCI, type of comorbid condition and number of comorbidities (31).

Controlling for stage of cancer

*North America*

Seven *colon* cancer studies controlled for cancer stage by using multivariate regression (5, 8, 16, 20, 24, 27, 29), five studies only reported one cancer stage (4, 9, 10, 13, 22), and one study reported outcome data in sub-samples by cancer stage (21).

Of those *rectal* cancer studies controlling for cancer stage, five studies controlled for cancer stage by using multivariate regression (2, 17, 19, 24, 27), and two studies used multivariate analysis with propensity score matching (12, 15). The remaining two studies only reported one cancer stage (3, 11).

*Europe*

Of those *colon* cancer studies controlling for cancer stage, two studies used multivariate regression (6, 7), and one used univariate analysis using cancer stage (26). One study only reported one cancer stage (18), one study reported outcome data in sub-samples by cancer stage (1), and the remaining three studies did not report the methods of cancer stage control (23, 25, 30).

Of those *rectal* cancer studies controlling for cancer stage, two studies controlled for cancer stage by using multivariate regression (7, 28), one study used univariate analysis (26), and one study reported outcome data in sub-samples by cancer stage (1). The remaining three studies did not report methods of cancer stage control (14, 25, 30).

*Oceania*

The *colon* cancer study controlled for cancer stage by using multivariate regression (31).

**Systematic search strategy**

| **#** | **Search terms** |
| --- | --- |
| 5 | ((gastr* or stomach or ?esophag* or colorectal or colon or colonic or rectum or rectal or lung* or  pulmonary or bronchi*) adj3 (cancer* or neopla* or tumor* or tumour* or carcinoma* or adenocarcinoma* or small cell or squamous)).ti,ab,kw. |
| 7 | exp survival analysis/ |
| 8 | exp "Severity of Illness Index"/ |
| 9 | Tumor Burden/ |
| 10 | ((disease* or ill*) adj2 (sever* or burden or outcome* or length or long or surviv* or incidence or duration   or predict* or prognosis)).ti,ab,kw. |
| 11 | proportional hazards models/ |
| 12 | "length of stay"/ |
| 13 | length of stay.ti,ab,kw. |
| 14 | Time-to-Treatment/ |
| 15 | (treatment adj2 (outcome* or length or long or duration or allocate* or respon* or delay*)).ti,ab,kw. |
| 16 | exp treatment outcome/ |
| 17 | exp Hospitalization/ |
| 18 | hospitali?ation.ti,ab,kw. |
| 19 | (("accident and emergency" or "a and e" or "a & e" or hospital*) adj3 (admit or admission* or admittance  or entry or enter or access)).ti,ab,kw. |
| 20 | exp Mortality/ |
| 21 | kaplan-meier estimate/ |
| 22 | ((mortality or death* or survival or survive) adj2 (estimate* or rate* or amount*)).ti,ab,kw. |
| 23 | Kaplan meier.ti,ab,kw. |
| 24 | Health Resources/ |
| 25 | (resource adj2 (use* or utili?ation)).ti,ab,kw. |
| 26 | "Quality of Life"/ |
| 27 | exp Health Care Costs/ |
| 28 | "Value of Life"/ |
| 29 | quality-adjusted life years/ |
| 30 | ((instrument or instruments) adj3 quality of life).ab. |
| 31 | quality of life.ti,kf. |
| 32 | quality adjusted life.ti,ab,kf. |
| 33 | (qaly* or qald* or qale* or qtime* or life year or life years).ti,ab,kf. |
| 34 | Economics/ |
| 35 | exp "Costs and Cost Analysis"/ |
| 36 | (economic* or cost or costs or costly or costing or price or prices or pricing or pharmacoeconomic* or  pharmaco-economic* or expenditure or expenditures or expense or expenses or financial or finance or  finances or financed).ti,kf. |
| 37 | exp Economics, Hospital/ |
| 38 | Economics, Nursing/ |
| 39 | Economics, Medical/ |
| 40 | Economics, Pharmaceutical/ |
| 41 | exp Budgets/ |
| 42 | (economic* or cost or costs or costly or costing or price or prices or pricing or pharmacoeconomic* or  pharmaco-economic* or expenditure or expenditures or expense or expenses or financial or finance or  finances or financed).ab. /freq=2 |
| 43 | (cost* adj2 (effective* or utilit* or benefit* or minimi* or analy* or outcome or outcomes)).ab,kf. |
| 44 | or/7-43 |
| 45 | (co-morbid* or comorbid* or multi-morbid* or multimorbid*).ti,ab,kw. |
| 46 | exp Comorbidity/ |
| 47 | ((multiple or chronic) adj (ill* or disease* or condition* or syndrom* or disorder*)).ti,ab,kw. |
| 48 | (multidisease* or multi-disease*).ti,ab,kw. |
| 49 | ((coocur* or co-ocur* or coexist* or co-exist* or multipl* or concord* or discord*) adj3 (disease* or ill* or  care or condition? or disorder* or health* or medication* or symptom* or syndrom*)).ti,ab,kw. |
| 50 | or/45-49 |
| 51 | 6 and 44 and 50 |

**Original Study Protocol on PROSPERO (CRD42021227087)**

**Impact of comorbidity on clinical pathways for cancer patients: a systematic review**

**Introduction**

The world’s population is ageing, leading to an increase in the prevalence of multiple co-occurring health conditions (32). With the majority of cancer patients diagnosed after the age of 65, there is a substantial proportion of patients dealing with cancer alongside one or more comorbidities (33). The presence of these additional chronic conditions has been found to have an adverse impact on survival among cancer patients, both in terms of their cancer prognosis, and the increased risk of non-cancer mortality (34, 35). Thus, as a result, the complex relationship between cancer and comorbidity has warranted further attention.

There is a growing body of evidence relating to the impact of comorbidity on all aspects of the clinical pathway, including diagnosis, treatment and survival (36). The 2019 Renzi review demonstrated that, depending on the previously diagnosed chronic condition(s) present in the patient, diagnosis can be either expedited or impeded at multiple stages due to screening, help-seeking behaviour, emergency presentation and referrals (37). Delayed diagnosis commonly occurs due to the comorbidity masking symptoms and/or competing for clinical attention, often resulting in poorer survival. Additionally, studies consistently find that cancer patients with comorbidity are less likely to receive curative treatment than those without comorbidity (38-42). This research also extends to separate parts of the clinical pathway, such as the differential length of hospital stay after treatment (43). Multiple studies have since been presented to explain these differences. For example, vignette-based studies on clinicians, surgeons and oncologists show they are less likely to refer and recommend treatment, possibly due to arguments surrounding the reduced effectiveness and increased toxicity in comorbid patients (44-46).

Despite these numerous studies and explanations, there has yet to be a review conducted on the overall heterogeneity of clinical pathways for cancer patients directly relating to the presence of comorbidities. Current systematic reviews are limited to either a specific comorbidity and cancer combination or, singular interventions, such as differences in chemotherapy use. This review therefore aims to expand on the current literature by analysing differences in patterns of resource utilisation and treatment management in cancer patients with comorbidities compared to those without. This will allow a broader understanding of the clinical pathway as a whole.

**Objectives**

**Review objective**

The primary objective of this review is to identify differences in the clinical pathways of cancer patients due to the presence/ type of comorbidities. This will include the resource health care utilisation (e.g. hospital admissions), treatment management (e.g. the prevalence of surgery), as well as patient outcomes (e.g. mortality rates, health-related quality of life measure and utility values).

**Research questions**

1. Do the clinical pathways differ in cancer patients with comorbidity, compared to those without, after controlling for confounders (e.g. stage of diagnosis)?
2. If the clinical pathways identified in question 1 differ, are there different outcomes for cancer patient with, compared to without, comorbidity as a result?
3. If so, does health care utilisation also differ between these patients?

**Methods**

This protocol has been developed following the ‘Preferred reporting items for systematic reviews and meta-analysis protocols (PRISMA-P) 2015 statement’ (48).

**Eligibility criteria**

The following headings, based on the PICOS framework (population, intervention, comparison, outcome and study design), describe the inclusion and exclusion criteria with the addition of extra categories:

*Population*

Individuals aged 18 years or over at the time of their first diagnosis with colorectal, oesophageal, gastric or ovarian cancer. Any studies with further restrictions on patient characteristics (e.g. age groups, gender or ethnicity), will be excluded.

*Intervention*

All types of intervention will be included.

*Comparison*

Cancer patients that meet the population criteria with, compared to without, additional chronic conditions.

*Study Outcomes*

The measures to be included are:

- Type of treatment
- Treatment outcomes
- Health related quality of life
- Health care resource use
- Costs

*Study design*

Any study design will be included.

*Language*

Only English language studies will be included.

*Data restrictions*

Only studies published from January 2000 will be included.

**Search strategy and information sources**

Text words and index terms based on the eligibility criteria have been used to form a systematic multi-string strategy. This search will be conducted to retrieve studies from the published literature in PudMed (Medline) and Embase electronic databases.

**Data management**

EndNote version x9.2 will be utilised to store, remove duplicates and screen the titles and abstracts of the citations downloaded from the literature searches.

**Selection process**

In the initial stage of screening, the eligibility of titles and abstracts will be assessed, with those failing to meet the criteria being excluded. The full texts of the remaining citations will be retrieved for the second stage of screening in which the eligibility criteria will be applied. Two reviewers will make this assessment to allow discrepancies to be discussed and resolved by consensus. The results of the selection process will be documented in a PRISMA flow diagram, including the reasons for the exclusions of studies.

**Data extraction**

Data will be extracted from the included studies using a Microsoft Excel based data extraction template. This will allow for a standardised and consistent method of extraction across all studies, including the noting of when data is “not applicable” for any pre-specified fields. A second reviewer will check the obtained data for its accuracy and completeness allowing any discrepancies to be discussed and resolved by consensus. A summary of the extracted data, collated in Microsoft Excel, will be presented in tables in Microsoft Word where necessary.

**Data items**

The proposed variables for which data will be sought are listed in Table 1. This list will be updated upon the review of included studies as additional variables that may be relevant to the analysis are identified.

Table 1. Overview of expected variables to be extracted from included studies

| **Variable** | **Description** |
| --- | --- |
| **Study Characteristics** | |
| Author | Name of author |
| Year | Year of publication |
| Title | Article title |
| Journal | Journal title |
| Study design | Study type |
| Country | Country of study and populations |
| **Participant characteristics and Interventions considered** | |
| Population | Description of population including; number, age, and sex |
| Cancer | Description of cancer site(s) and stage(s) |
| Chronic conditions | Description of presence/absence of co-morbidities possibly including an index score or aggregate number |
| Interventions compared in cost effective analysis | What interventions were compared? |
| **Methods** | |
| Analysis | Describe the methods used to analyse the data |
| Confounders | What confounders are controlled for in these analyses (eg stage of diagnosis, age, sex etc)? |
| Exploration of heterogeneity | Are methods used to explore subjects heterogeneity eg subgoup analyses? |
| Cancer and comorbidities | Is the management of cancer and comorbidities explored in equal depth within the studies (in primary care, secondary care or emergency treatments)? |
| Model type | What models were used to extrapolate from the trial ends point to policy relevant horizon? |
| **Clinical pathway** | |
| Treatment characteristics | Description of the treatment(s) (types, dose, and delays) and the number/proportion of the population receiving them, sequence of treatments |
| Treatment outcomes | Description of mortality rates, complications, patient survival and/or hazard ratios |
| Health care resource use | Description of resource use by population in primary care, secondary care, day patients, and A&E including hospital admissions and length of stay, palliative care, and end of life care in hospices |
| Health-related quality of life measures | Descriptions of health related quality of life measures and values |
| Cost analysis | Description of economic analysis, and utilities including hospital, palliative and hospice care, along with out-of-pocket costs |

**Quality assessment**

All studies included in this systematic review will be assessed regarding their risk of bias and methodological quality. This will be conducted by two reviewers to facilitate the discussion of any discrepancies which will be resolved by consensus. The outcomes from the quality assessment will be summarised in tables and/or graphically. Studies deemed to be of low quality will still be included in the review so as to discuss the limitations of their analysis.

Methodological Quality checklists proposed by the NHS Centre for Reviews and Dissemination will be used to appraise study quality, eg CASP checklists for cost-effectiveness/ cost-utility studies alongside RCTs eg https://casp-uk.net/casp-tools-checklists/ and Philips checklist of modelled studies.

**Data synthesis**

Study findings will be qualitatively summarised in a narrative synthesis. Initially, the self-reported strengths and weaknesses of each study will be reviewed and critiqued. Subsequently, the authors of this review will undertake their own critique of the studies focusing on the following elements:

1. Methods
   1. What methods have been used to analyse differences and what confounders have been controlled for in these analyses?
   2. Is the management of cancer and comorbidities explored in equal depth across the studies?
   3. Is patient heterogeneity explored within the analyses?
2. What are the differences in the outcomes of the clinical pathway (such as patient survival, costs and health-related quality of life measures) depending on comorbidity?
3. Are there any gaps in the evidence base for any parts of the clinical pathway of cancer, for the comorbidities considered?
4. Does the resource use in primary, secondary or emergency services differ in cancer patients with comorbidities, compared to those without, after controlling for confounders (eg stage of diagnosis, age and sex)?

**References**

1. van Eeghen EE, Bakker SD, van Bochove A, Loffeld RJ. Impact of age and comorbidity on survival in colorectal cancer. Journal of Gastrointestinal Oncology. 2015;6(6):605-12.

2. Emile SH, Horesh N, Freund MR, Garoufalia Z, Gefen R, Silva-Alvarenga E, et al. A National Cancer Database analysis of the predictors of unplanned 30-day readmission after proctectomy for rectal adenocarcinoma: The CCF RETURN-30 Score. Surgery. 2023;173(2):342-9.

3. Freund MR, Horesh N, Emile SH, Garoufalia Z, Gefen R, Wexner SD. Predictors and outcomes of positive surgical margins after local excision of clinical T1 rectal cancer: A National Cancer Database analysis. Surgery. 2023;173(6):1359-66.

4. Reif de Paula T, Keller DS. A national evaluation of adjuvant chemotherapy in pT4N0M0 colon cancer from the National Cancer Database. J Natl Cancer Inst. 2023;115(12):1616-25.

5. Abdel-Rahman O, Tang PA, Koski S. Hospitalizations among early-stage colon cancer patients receiving adjuvant chemotherapy: a real-world study. Int J Colorectal Dis. 2021;36(9):1905-13.

6. Clouth FJ, Moncada-Torres A, Geleijnse G, Mols F, van Erning FN, de Hingh IHJT, et al. Heterogeneity in Quality of Life of Long-Term Colon Cancer Survivors: A Latent Class Analysis of the Population-Based PROFILES Registry. The oncologist. 2021;26(3):e492-e9.

7. Kellokumpu I, Kairaluoma M, Mecklin J-P, Kellokumpu H, Vayrynen V, Wirta E-V, et al. Impact of Age and Comorbidity on Multimodal Management and Survival from Colorectal Cancer: A Population-Based Study. Journal of clinical medicine. 2021;10(8).

8. Simon HL, Reif de Paula T, Spigel ZA, Keller DS. N1c colon cancer and the use of adjuvant chemotherapy: a current audit of the National Cancer Database. Colorectal disease : the official journal of the Association of Coloproctology of Great Britain and Ireland. 2021;23(3):653-63.

9. Mukkamalla SKR, Huynh DV, Somasundar PS, Rathore R. Adjuvant Chemotherapy and Tumor Sidedness in Stage II Colon Cancer: Analysis of the National Cancer Data Base. Frontiers in oncology. 2020;10:568417.

10. Wegner RE, Abel S, Monga D, Raj M, Finley G, Nosik S, et al. Utilization of Adjuvant Radiotherapy for Resected Colon Cancer and Its Effect on Outcome. Ann Surg Oncol. 2020;27(3):825-32.

11. Concors SJ, Vining CM, Saur NM, Roses RE, Paulson EC. Combined Proctectomy and Hepatectomy for Metastatic Rectal Cancer Should be Undertaken with Caution: Results of a National Cohort Study. Ann Surg Oncol. 2019;26(12):3972-9.

12. Wegner RE, Hasan S, Renz PB, Raj MS, Monga DK, Finley GG, et al. Definitive Chemoradiation for Rectal Cancer: Is There a Role for Dose Escalation? A National Cancer Database Study. Dis Colon Rectum. 2019;62(11):1336-43.

13. Xu Z, Becerra AZ, Fleming FJ, Aquina CT, Dolan JG, Monson JR, et al. Treatments for Stage IV Colon Cancer and Overall Survival. J Surg Res. 2019;242:47-54.

14. El Amrani M, Clement G, Lenne X, Rogosnitzky M, Theis D, Pruvot FR, et al. The Impact of Hospital Volume and Charlson Score on Postoperative Mortality of Proctectomy for Rectal Cancer: A Nationwide Study of 45,569 Patients. Ann Surg. 2018;268(5):854-60.

15. Wegner RE, Abel S, White RJ, Horne ZD, Hasan S, Kirichenko AV. Trends in intensity-modulated radiation therapy use for rectal cancer in the neoadjuvant setting: a National Cancer Database analysis. Radiation oncol. 2018;36(4):276-84.

16. Flemming JA, Nanji S, Wei X, Webber C, Groome P, Booth CM. Association between the time to surgery and survival among patients with colon cancer: A population-based study. Eur J Surg Oncol. 2017;43(8):1447-55.

17. Shahab D, Gabriel E, Attwood K, Ma WW, Francescutti V, Nurkin S, et al. Adjuvant Chemotherapy Is Associated With Improved Overall Survival in Locally Advanced Rectal Cancer After Achievement of a Pathologic Complete Response to Chemoradiation. Clin Colorectal Cancer. 2017;16(4):300-7.

18. van den Broek CBM, Puylaert C, Breugom AJ, Bastiaannet E, de Craen AJM, van de Velde CJH, et al. Administration of adjuvant chemotherapy in older patients with Stage III colon cancer: an observational study. Colorectal Disease. 2017;19(10):O358-O64.

19. Xu Z, Mohile SG, Tejani MA, Becerra AZ, Probst CP, Aquina CT, et al. Poor compliance with adjuvant chemotherapy use associated with poorer survival in patients with rectal cancer: An NCDB analysis. Cancer. 2017;123(1):52-61.

20. Becerra AZ, Berho ME, Probst CP, Aquina CT, Tejani MA, Gonzalez MG, et al. Variation in Hospital-Specific Rates of Suboptimal Lymphadenectomy and Survival in Colon Cancer: Evidence from the National Cancer Data Base. Ann Surg Oncol. 2016;23(Suppl 5):674-83.

21. Chandhoke G, Wei X, Nanji S, Biagi J, Peng Y, Krzyzanowska M, et al. Patterns of Referral for Adjuvant Chemotherapy for Stage II and III Colon Cancer: A Population-Based Study. Ann Surg Oncol. 2016;23(8):2529-38.

22. Hsieh MC, Thompson T, Wu XC, Styles T, O'Flarity MB, Morris CR, et al. The effect of comorbidity on the use of adjuvant chemotherapy and type of regimen for curatively resected stage III colon cancer patients. Cancer Med. 2016;5(5):871-80.

23. Cakir H, Heus C, Verduin WM, Lak A, Doodeman HJ, Bemelman WA, et al. Visceral obesity, body mass index and risk of complications after colon cancer resection: A retrospective cohort study. Surgery. 2015;157(5):909-15.

24. Yeo H, Niland J, Milne D, ter Veer A, Bekaii-Saab T, Farma JM, et al. Incidence of minimally invasive colorectal cancer surgery at National Comprehensive Cancer Network centers. J Natl Cancer Inst. 2015;107(1):362.

25. Ostenfeld EB, Norgaard M, Thomsen RW, Iversen LH, Jacobsen JB, Sogaard M. Comorbidity and survival of Danish patients with colon and rectal cancer from 2000-2011: a population-based cohort study. Clin Epidemiol. 2013;5(Suppl 1):65-74.

26. Gooiker GA, Dekker JW, Bastiaannet E, van der Geest LG, Merkus JW, van de Velde CJ, et al. Risk factors for excess mortality in the first year after curative surgery for colorectal cancer. Ann Surg Oncol. 2012;19(8):2428-34.

27. Bilimoria KY, Ko CY, Tomlinson JS, Stewart AK, Talamonti MS, Hynes DL, et al. Wait times for cancer surgery in the United States: trends and predictors of delays. Ann Surg. 2011;253(4):779-85.

28. Gort M, Otter R, Plukker JT, Broekhuis M, Klazinga NS. Actionable indicators for short and long term outcomes in rectal cancer. European Journal of Cancer. 2010;46(10):1808-14.

29. Hines RB, Chatla C, Bumpers HL, Waterbor JW, McGwin G, Jr., Funkhouser E, et al. Predictive capacity of three comorbidity indices in estimating mortality after surgery for colon cancer. Journal of Clinical Oncology. 2009;27(26):4339-45.

30. Iversen LH, Norgaard M, Jacobsen J, Laurberg S, H.T SL. The impact of comorbidity on survival of danish colorectal cancer patients from 1995 to 2006 ' a population-based cohort study. Diseases of the Colon and Rectum. 2009;52(1):71-8.

31. Sarfati D, Hill S, Blakely T, Robson B, Purdie G, Dennett E, et al. The effect of comorbidity on the use of adjuvant chemotherapy and survival from colon cancer: a retrospective cohort study. BMC Cancer. 2009;9:116.

32. Yancik R, Havlik RJ, Wesley MN, Ries L, Long S, Rossi WK, et al. Cancer and comorbidity in older patients: A descriptive profile. Annals of Epidemiology. 1996;6(5):399-412.

33. Jemal A, Siegel R, Xu J, Ward E. Cancer statistics, 2010. CA: a cancer journal for clinicians. 2010;60(5):277-300.

34. Tammemagi CM, Neslund‐Dudas C, Simoff M, Kvale P. Impact of comorbidity on lung cancer survival. Int J Cancer. 2003;103(6):792-802.

35. Baade PD, Fritschi L, Eakin EG. Non-Cancer Mortality among People Diagnosed with Cancer (Australia). Cancer Causes & Control. 2006;17(3):287-97.

36. Søgaard M, Thomsen R, Bossen K, Sørensen H, Nørgaard M. The impact of comorbidity on cancer survival: a review. Clin Epidemiol. 2013;5:3-29.

37. Renzi C, Kaushal A, Emery J, Hamilton W, Neal RD, Rachet B, et al. Comorbid chronic diseases and cancer diagnosis: disease-specific effects and underlying mechanisms. Nature Reviews Clinical Oncology. 2019;16(12):746-61.

38. Ramsey SD, Howlader N, Etzioni RD, Donato B. Chemotherapy Use, Outcomes, and Costs for Older Persons With Advanced Non–Small-Cell Lung Cancer: Evidence From Surveillance, Epidemiology and End Results–Medicare. Journal of Clinical Oncology. 2004;22(24):4971-8.

39. Koppie TM, Serio AM, Vickers AJ, Vora K, Dalbagni G, Donat SM, et al. Age‐adjusted Charlson comorbidity score is associated with treatment decisions and clinical outcomes for patients undergoing radical cystectomy for bladder cancer. Cancer: Interdisciplinary International Journal of the American Cancer Society. 2008;112(11):2384-92.

40. Sarfati D, Hill S, Blakely T, Robson B, Purdie G, Dennett E, et al. The effect of comorbidity on the use of adjuvant chemotherapy and survival from colon cancer: a retrospective cohort study. BMC Cancer. 2009;9(1):116.

41. Rodrigues G, Sanatani M. Age and Comorbidity Considerations Related to Radiotherapy and Chemotherapy Administration. Seminars in Radiation Oncology. 2012;22(4):277-83.

42. Mellemgaard A, Lüchtenborg M, Iachina M, Jakobsen E, Green A, Krasnik M, et al. Role of Comorbidity on Survival after Radiotherapy and Chemotherapy for Nonsurgically Treated Lung Cancer. Journal of Thoracic Oncology. 2015;10(2):272-9.

43. Extermann M. Measurement and impact of comorbidity in older cancer patients. Critical Reviews in Oncology/ Hematology. 2000;35(3):181-200.

44. Keating NL, Landrum MB, Klabunde CN, Fletcher RH, Rogers SO, Doucette WR, et al. Adjuvant Chemotherapy for Stage III Colon Cancer: Do Physicians Agree About the Importance of Patient Age and Comorbidity? Journal of Clinical Oncology. 2008;26(15):2532-7.

45. Krzyzanowska M, Regan M, Powell M, Earle C, Weeks J. Impact of Patient Age and Comorbidity on Surgeon Versus Oncologist Preferences for Adjuvant Chemotherapy for Stage III Colon Cancer. Journal of the American College of Surgeons. 2009;208(2):202-9.

46. Ring A, Harder H, Langridge C, Ballinger RS, Fallowfield LJ. Adjuvant chemotherapy in elderly women with breast cancer (AChEW): an observational study identifying MDT perceptions and barriers to decision making. Annals of oncology : official journal of the European Society for Medical Oncology. 2013;24(5):1211-9.

47. Lee L, Cheung WY, Atkinson E, Krzyzanowska MK. Impact of Comorbidity on Chemotherapy Use and Outcomes in Solid Tumors: A Systematic Review. Journal of Clinical Oncology. 2011;29(1):106-17.

48. Moher D, Shamseer L, Clarke M, Ghersi D, Liberati A, Petticrew M, et al. Preferred reporting items for systematic review and meta-analysis protocols (PRISMA-P) 2015 statement. Systematic Reviews. 2015;4(1):1.
