# Supplementary material for: Association between comorbidities and differences in treatment decisions and outcomes in patients with colon or rectal cancer: a systematic review
Source: BMJ Open. 2026 May 5;16(5):e096492. doi: 10.1136/bmjopen-2024-096492 (PMC13150882; doi:10.1136/bmjopen-2024-096492)
Supplement: Supplementary file 2 [file bmjopen-2024-096492.reviewer_comments.pdf]

PEER REVIEW HISTORY

BMJ Open publishes all reviews undertaken for accepted manuscripts. Reviewers are asked to complete a checklist review form and are provided with free text boxes to elaborate on their assessment. These free text comments are reproduced below.

ARTICLE DETAILS

Title (Provisional)

The association between comorbidities and differences in treatment decisions and outcomes in patients with colon or rectal cancer: a systematic review

Authors

Lloyd, Abigail Katie; Medina-Lara, Antonieta; Birch, Stephen; Wallis, Katharine; Spencer, Anne

---

VERSION 1 - REVIEW

**Reviewer**	**1**
**Name**	**Moore, Dirk**
**Affiliation**	**Rutgers, Biostatistics and Epidemiology**
**Date**	**04-Mar-2025**
**COI**	**None**

This manuscript presents a descriptive systematic analysis of 31 colon cancer studies and of factors affecting patient access and survival. The paper screening methods were comprehensive and appropriate. Since this manuscript is descriptive in nature, no statistical analyses were needed. However, the authors often refer to statistical methods used in the studies they are summarizing, as approrpriate.

**Reviewer**	**2**
**Name**	**Dack, Kyle**
**Affiliation**	**University of Bristol, MRC Integrative Epidemiology Unit**
**Date**	**06-Mar-2025**
**COI**	**None**

This systematic review is clear, well written, and the authors have done well to manage and synthesize so many results, which I know is a challenge. I do however have a few major concerns regarding the methods and reporting:

1. The results section focuses only on significant results. This makes it hard to understand the overall pattern of evidence. It would much more informative if the synthesis included all studies, e.g. “10 of 14 studies reported significant associations, and 4 found no association”. This also impacts the discussion, particularly the beginning which seems to focus on significant results and not comment on the overall strength of evidence.

2. The quality assessment tool had poor sensitivity (all scored 8+ out of 10 items)– and it seems very unlikely to me that from 31 studies, all are equally reliable. This is unfortunate because it means the synthesis cannot stratify results by high/low quality, which could have added reliability to the findings. I am not sure if much can be done about it, but at least this should be highlighted as a limitation in the discussion.

3. There are some important elements missing from the methods which I have commented on below, and I recommend the authors review the PRISMA checklist and elaboration document to find more details.

Further comments:

Abstract

1. I think the objectives and design have a high risk of being misinterpreted. The design states that studies were included which “evaluated associations between comorbidities and cancer”. Similarly, the objective begins with the intention to evaluate “associations between comorbidity and colon and rectal cancer”.

Many readers are likely to interpret this as evaluating how comorbidities affect the risk/odds of cancer, which is not a part of this study – colon and rectal cancer patients are just the population of interest. The objective would be more accurately described as “To systematically evaluate associations between comorbidities and differences in treatment decisions, outcomes,health-related quality of life (HRQoL), health-care resource utilisation and costs, in patients with colon or rectal cancer”. I suggest clarifying this throughout the paper to avoid any confusion.

Introduction

3. The introduction is clear, sets the context well and is supported by up to date references. However, I suggest that lines 61-62 are expanded on. This sentence states that two prior systematic reviews of comorbidities exist – but are these specific to colon/rectal cancer? Which outcomes did these reviews cover, and what was the general conclusion? I think more detail is necessary because the next sentence talks about an evidence gap – but it is not really clear what this gap is, or how this new review will fit with the past reviews.

Methods

4. The study selection subheading currently contains quite a lot of information not really relevant to the inclusion/exclusion criteria. It would be clearer if this was divided into study design – search strategy – inclusion/exclusion criteria – screening process, or similar.

5. The search strategy lacks details required for PRISMA item 7 (which is currently not marked as completed in the checklist). The main text should include the general approach – the main keywords, date ranges, language restrictions etc, with the complete search terms for both databases in a supplementary file.

6. Line 87-88. This sentence seems to imply that all studies were screened by abstract, and full texts were only checked if uncertain. Could the authors confirm this? It is not consistent with Figure 1 which shows a two stage screening process.

Results

7. Figure 1. The exclusion reasons should be consistent with the exclusion criteria in the methods. Currently there isn't a clear list in the methods, I suggest adding this and checking to make sure it is consistent with the flow chart.

8. Quality assessment: Something which I think could be a limitation of this review is that the quality assessment does not seem very sensitive. Looking at tables S1/S2, every study scored 8 or more out of 10 criteria. It seems very unlikely to me that all these studies are really high quality. For example, there may be 2-3 key confounders, which some studies failed to measure or adjust for. Poor modelling strategies, attrition and selection bias also tend to be common risks in these types of study. I would suggest discussing this in the discussion, whether the JBI was sensitive enough.

9. Line 119. It would be helpful for readers to know what the main confounders are in the included analyses.

10. Lines 125-129. The references nested in the parentheses make this section hard to read, and I suggest it would look better if they were moved to the end of the sentences. The authors understandably wish to attribute each reference to a specific included study, but this is already achieved in Table 1.

11. Table 1. The intervention column is unclear – these are all observational studies, how could there be an intervention? This may need more explanation in the text or methods. Would "comparison" be more accurate?

12. Table 1. The age column – I don't think "age" is needed in the individual cells – it takes up space. Some studies such as the first contain two numbers, what do these mean?

13. Outcome results. The way results are summarised on line 145 ("total significant results / total studies") is very helpful. It would help to do this for all reported outcomes, e.g. the next line 147, reports delays to treatment. Significant findings are reported from two studies. But from how many studies in total? The reader cannot understand the overall strength of evidence without this information.

Discussion

14. Lines 255-267. I don't think it is informative for the denominator to be all studies of colon/rectal cancer. It is more informative to report the number significant out of the total number of studies for that particular outcome.

The numerator also seems inaccurate? E.g The first, "1/22" I assume means 1 study reported a significant difference in treatment delay out of 22 total studies of colon cancer. But looking at the results, I see in North America two studies reported significant differences (line 147).

15. I think that generally the results and discussion are focused very much on significant results. But as this is a synthesis of all studies, the focus should be more on the overall strength of evidence. It is hard for me to check if all of the conclusions are accurate because non-significant results are currently not reported in the results.

**Reviewer**	**3**
**Name**	**Araújo-Filho, Irami**
**Affiliation**	**UFRN, SURGERY**
**Date**	**30-Jul-2025**
**COI**	**None**

I would like to commend the authors for this well-executed and timely systematic review. The manuscript addresses an important clinical and policy-relevant topic—the impact of comorbidities on treatment decisions and outcomes in patients with colon and rectal cancer in high-income countries. The research question is clearly defined, the methodology is rigorous, and the narrative synthesis is comprehensive and well-structured. The use of PRISMA methodology, inclusion of only high-income countries to control for health system variability, and restriction to widely used comorbidity indices all contribute to the robustness of the review.

That said, I recommend minor revisions to further strengthen the manuscript:

Clarify the limitations more explicitly in the discussion and conclusion, especially regarding the scarcity of data on health-related quality of life (HRQoL), healthcare costs, and resource utilization. These gaps are important for policy implications and future research priorities.

Ensure the inclusion of the full PRISMA flow diagram and search strategy details (e.g., search terms, filters, date of last search) in either the main manuscript or supplementary materials to improve transparency and reproducibility.

Standardize the terminology and abbreviations across the text when referring to comorbidity indices (e.g., Charlson, Charlson-Deyo, ACCI, mCCI), and consider adding a glossary or footnote where these are explained concisely.

Improve minor issues with language clarity and redundancy, particularly in the discussion section, to enhance overall readability. While the English is of a high standard, a final proofreading pass would be helpful.

Ensure figures (particularly Figures 2 and 3) are clear, well-labeled, and properly referenced within the text. If they are included in the Supplementary Materials, this should be explicitly mentioned.

Overall, this is an important and well-constructed review that contributes to the literature on cancer outcomes and comorbidity. With these minor revisions, the manuscript will be suitable for publication.

However, I recommend minor revisions to enhance the clarity and utility of the manuscript:

Expand the discussion of limitations, particularly regarding the limited data on HRQoL, healthcare resource utilization, and costs. These are key gaps that affect the strength of conclusions and the scope of recommendations.

Include complete search strategy details, including search terms, date ranges, and filters used. If placed in Supplementary Materials, indicate this clearly.

Standardize terminology and abbreviations for comorbidity indices throughout the manuscript (e.g., CCI, mCCI, ACCI, Charlson-Deyo), and consider adding a clarifying note or footnote.

Ensure final proofreading for style and clarity, especially in the discussion section. Although the manuscript is written in fluent English, some redundancies and minor phrasing adjustments would improve flow.

Ensure that Figures 2 and 3 are clear and well integrated with the results section. If they appear only in the supplementary file, indicate this explicitly in the main text.

This manuscript adds significant value to the literature and is suitable for publication following these minor improvements.

VERSION 1 - AUTHOR RESPONSE

Comment	
I would like to commend the authors for this well-executed and timely systematic review. The manuscript addresses an important clinical and policy-relevant topic—the impact of comorbidities on treatment decisions and outcomes in patients with colon and rectal cancer in high-income countries. The research question is clearly defined, the methodology is rigorous, and the narrative synthesis is comprehensive and well-structured. The use of PRISMA	We would like to thank the reviewers for their commendations.

methodology, inclusion of only high-income countries to control for health system variability, and restriction to widely used comorbidity indices all contribute to the robustness of the review.	
Reviewer: 2<br>1. The results section focuses only on significant results. This makes it hard to understand the overall pattern of evidence. It would much more informative if the synthesis included all studies, e.g. "10 of 14 studies reported significant associations, and 4 found no association". This also impacts the discussion, particularly the beginning which seems to focus on significant results and not comment on the overall strength of evidence.<br>13. Outcome results. The way results are summarised on line 145 ("total significant results / total studies") is very helpful. It would help to do this for all reported outcomes, e.g. the next line 147, reports delays to treatment. Significant findings are reported from two studies. But from how many studies in total? The reader cannot understand the overall strength of evidence without this information.	We thank the reviewer for this comment and have amended the sections accordingly.<br>e.g.<br>For colon cancer studies, 5 in 13 studies explored survival and found associations between comorbidities and lower survival rates(19, 21, 22, 29, 30). For studies reporting differences, higher CCI scores were linked with lower overall survival and cancer-specific survival in one study(30), and non-significant lower overall survival in another study(29). Additionally, higher CD scores were associated with statistically significantly lower 5-year survival(21), and overall survival(19, 22).
2. The quality assessment tool had poor sensitivity (all scored 8+ out of 10 items)– and it seems very unlikely to me that from 31 studies, all are equally reliable. This is unfortunate because it means the synthesis cannot stratify results by high/low quality, which could have added reliability to the findings. I am not sure if much can be done about it, but at least this should be highlighted as a limitation in the discussion.<br><br>8. Quality assessment: Something which I think could be a limitation of this review is that the quality assessment does not seem very sensitive. Looking at tables S1/S2, every study scored 8 or more out of 10 criteria. It seems very unlikely to me that all these studies are really high quality. For example, there may be 2-3 key confounders, which	We thank the reviewer for this comment and have added a discussion of the tool's poor sensitivity to limitations. In particular we now write in the limitations section:<br>"Finally, a weakness of the review is that the quality assessment tool that we used was not very sensitive and all studies scored 8 or more out of the 10 point criteria. The Joanna Briggs Institute is a well-respected quality assessment tool but in this application determining whether the two groups were similar and recruited from the same population was often unclear, and could not easily to assessed in these types of studies."

some studies failed to measure or adjust for. Poor modelling strategies, attrition and selection bias also tend to be common risks in these types of study. I would suggest discussing this in the discussion, whether the JBI was sensitive enough.	
3. There are some important elements missing from the methods which I have commented on below, and I recommend the authors review the PRISMA checklist and elaboration document to find more details.	We have checked the PRISMA checklist and added more details to the paper accordingly.
Abstract<br>Abstract<br>1. I think the objectives and design have a high risk of being misinterpreted. The design states that studies were included which "evaluated associations between comorbidities and cancer". Similarly, the objective begins with the intention to evaluate "associations between comorbidity and colon and rectal cancer".<br>Many readers are likely to interpret this as evaluating how comorbidities affect the risk/odds of cancer, which is not a part of this study – colon and rectal cancer patients are just the population of interest. The objective would be more accurately described as "To systematically evaluate associations between comorbidities and differences in treatment decisions, outcomes,health-related quality of life (HRQoL), health-care resource utilisation and costs, in patients with colon or rectal cancer". I suggest clarifying this throughout the paper to avoid any confusion.	We have amended and added the text<br><br>"To systematically evaluate associations between comorbidities and differences in treatment decisions, outcomes,health-related quality of life (HRQoL), health-care resource utilisation and costs, in patients with colon or rectal cancer"
Introduction<br>3. The introduction is clear, sets the context well and is supported by up to date references. However, I suggest that lines 61-62 are expanded on. This sentence states that two prior systematic reviews of comorbidities exist – but are these specific to colon/rectal cancer? Which outcomes did these reviews cover, and what was the general conclusion? I think more detail is necessary because the next sentence talks about an evidence gap – but it is not really	Thank you we have expanded the lines 61-62 and adding the following text:<br>In colon and rectal cancer research, previous systematic reviews on comorbidities generally focus on a singular cancer intervention for example, adjuvant chemotherapy in stage III colon cancer patients (11), or on one comorbidity, for example diabetes (12).

clear what this gap is, or how this new review will fit with the past reviews.	
Methods<br>4. The study selection subheading currently contains quite a lot of information not really relevant to the inclusion/exclusion criteria. It would be clearer if this was divided into study design – search strategy – inclusion/exclusion criteria – screening process, or similar.<br><br>5. The search strategy lacks details required for PRISMA item 7 (which is currently not marked as completed in the checklist). The main text should include the general approach – the main keywords, date ranges, language restrictions etc, with the complete search terms for both databases in a supplementary file.<br><br>6. Line 87-88. This sentence seems to imply that all studies were screened by abstract, and full texts were only checked if uncertain. Could the authors confirm this? It is not consistent with Figure 1 which shows a two stage screening process.<br><br>Reviewer 3<br>Ensure the inclusion of the full PRISMA flow diagram and search strategy details (e.g., search terms, filters, date of last search) in either the main manuscript or supplementary materials to improve transparency and reproducibility.	We have divided now the methods into study design – search strategy – inclusion/exclusion criteria – screening process.<br><br>PRISMA item 7<br><br>We have added the searches as a supplementary file.
Results<br>7. Figure 1. The exclusion reasons should be consistent with the exclusion criteria in the methods. Currently there isn't a clear list in the methods, I suggest adding this and checking to make sure it is consistent with the flow chart.	We have amended the methods accordingly and added all the exclusion criteria or failure to meet the inclusion criteria given in the PRISMA into the methods.
9. Line 119. It would be helpful for readers to know what the main confounders are in the included analyses.	We have added the following text<br>Most cohort studies clearly identified confounding variables (29/30), such as age, ethnicity, cancer stage, and the route to

10. Lines 125-129. The references nested in the parentheses make this section hard to read, and I suggest it would look better if they were moved to the end of the sentences. The authors understandably wish to attribute each reference to a specific included study, but this is already achieved in Table 1.	diagnosis and strategies to deal with them (28/30)<br><br>Lines 125-129 we have amended the references.
11. Table 1. The intervention column is unclear – these are all observational studies, how could there be an intervention? This may need more explanation in the text or methods. Would "comparison" be more accurate?<br><br>12. Table 1. The age column – I don't think "age" is needed in the individual cells – it takes up space. Some studies such as the first contain two numbers, what do these mean?	We have amended the column title.<br><br>For the age column, we have removed "age" from the cells as suggested.<br><br>If the cells in the age column contain more than one number (e.g. median age 63/77) this is when the study has divided the population into two groups and reported the median age for each group (e.g. one was 63 and the other 77).<br>We have added a footnote to Table 1 and 2, i.e..<br><br>* Age values are given as mean or median for the study, or for each subgroup (e.g., subgroup 1/subgroup 2).
Reviewer 3<br>Standardize the terminology and abbreviations across the text when referring to comorbidity indices (e.g., Charlson, Charlson-Deyo, ACCI, mCCI), and consider adding a glossary or footnote where these are explained concisely.	We have standardised the terminology across the text and in the tables and have added details to the legends on Figures 2 and 3, e.g. The legends of Figure 2 and 3 report that the results are divided into sections based upon the comorbidity measure used: Charlson Comorbidity Index (CCI), age-adjusted CCI (ACCI), modified version of CCI (mCCI), Charlson-Deyo Index (CD), types of comorbid conditions (CT), the number of comorbidities (N), and the presence of comorbidities (P).
Discussion<br>14. Lines 255-267. I don't think it is informative for the denominator to be all studies of colon/rectal cancer. It is more informative to report the number significant out of the total number of studies for that particular outcome.<br>The numerator also seems inaccurate? E.g The first, "1/22" I assume means 1 study reported a significant difference in treatment delay out of 22 total studies of colon cancer. But looking at the results, I see in North	We have now adjusted the denominator to be all studies that reported on that outcome for colon and rectal cancer respectively and added the following text:<br>"This section gives an overview of the principle findings of the review, and reports in paretheses the number of studies reporting significant differences out of the total number of studies assessing each outcome, i.e. ("total significant results / total studies measuring each outcome")."

America two studies reported significant differences (line 147).<br><br>15. I think that generally the results and discussion are focused very much on significant results. But as this is a synthesis of all studies, the focus should be more on the overall strength of evidence. It is hard for me to check if all of the conclusions are accurate because non-significant results are currently not reported in the results.	
Reviewer 3<br><br>Discussion<br>Clarify the limitations more explicitly in the discussion and conclusion, especially regarding the scarcity of data on health-related quality of life (HRQoL), healthcare costs, and resource utilization. These gaps are important for policy implications and future research priorities.	We have clarified the limitations more explicitly.
Discussion<br>Improve minor issues with language clarity and redundancy, particularly in the discussion section, to enhance overall readability. While the English is of a high standard, a final proofreading pass would be helpful.	We thank the reviewer and have undertaken a further proof reading of the document.

VERSION 2 - REVIEW

**Reviewer**	**2**
**Name**	**Dack, Kyle**
**Affiliation**	**University of Bristol, MRC Integrative Epidemiology Unit**
**Date**	**14-Feb-2026**
**COI**	

Thank you to the authors for their responses to my comments and suggestions. The methods and the overall pattern of evidence are now clear, and I would recommend the paper for publication.
